# Polytrauma Cases in the Emergency Department of a Community Hospital in Croatia

**DOI:** 10.3390/jpm15100483

**Published:** 2025-10-10

**Authors:** Ivana Herak, Ante Mihanović, Andrea Cvitković Roić, Anita Lukic, Sonja Obranić, Denis Grgurović, Ines Kalinić, Valentina Vincek, Ivo Dumić-Čule, Marijana Neuberg

**Affiliations:** 1 Department of Nursing, University North, 104 Brigade 3, 42000 Varazdin, Croatia; ivherak@unin.hr (I.H.); lukic.anita@yahoo.com (A.L.); sobranic@unin.hr (S.O.); inekalinic@unin.hr (I.K.); vavincek@unin.hr (V.V.); mneuberg@unin.hr (M.N.); 2 Split-Dalmatia County Pharmacy, Dugopoljska 3, 21204 Dugopolje, Croatia; ante.mihanovic@yahoo.com; 3 Helena Clinic for Pediatric Medicine, Kneza Branimira 71, 10000 Zagreb, Croatia; andreack@workmail.com; 4 Faculty of Medicine, Josip Juraj Strossmayer University of Osijek, Josipa Huttlera 4, 31000 Osijek, Croatia; 5 Faculty of Medicine, University of Rijeka, Braće Branchetta 20/1, 51000 Rijeka, Croatia; 6 Varazdin General Hospital, I. Meštrovića bb., 42000 Varazdin, Croatia; 7 Bjelovar University of Applied Sciences, Trg Eugena Kvaternika 4, 43000 Bjelovar, Croatia; 8 Trauma Department, Čakovec County Hospital, I. G. Kovačića, 1E, 40000 Cakovec, Croatia; dgrgurov@gmail.com

**Keywords:** polytrauma, emergency service, multidisciplinary care team, injury severity score

## Abstract

**Background**: The purpose of this study was to quantify the incidence of polytrauma cases at a single-center county hospital in Croatia and evaluate the therapeutic approaches currently in use. **Methods**: Patient data for 54 individuals diagnosed with polytrauma between 2019 and 2022 were retrospectively reviewed using the hospital’s medical records system. The analysis encompassed several aspects, including injury mechanisms, injury timing, Glasgow Coma Scale scores, alcohol levels, therapies, triage classifications, and hospital stay durations. **Results**: In this study, patient age was not significantly associated with clinical presentation, treatment approach, or outcomes. However, gender showed significant associations with GCS, triage category, and discharge status, with female patients presenting more frequently with severe impairment (GCS 3–8) and higher triage urgency. Blood alcohol levels were more frequently elevated in male patients but showed no association with clinical severity or outcomes. Additionally, lower GCS scores were significantly linked to poorer outcomes, including higher in-hospital mortality, while surgical intervention was associated with longer hospital stays. **Conclusions**: Collectively, gender and level of consciousness significantly influenced triage urgency and outcomes, highlighting the need for targeted prevention and management strategies.

## 1. Introduction

Injuries represent a significant global public health issue, affecting individuals across all age groups and socioeconomic backgrounds. Numerous factors, such as falls, sports, violence, accidents, and occupational hazards, contribute to these injuries. In addition to causing physical discomfort and disability, injuries can have a significant psychological and social impact on those affected, as well as their family members. The required medical interventions range from simple first aid to complex surgery and prolonged rehabilitation, depending on the severity of the injury [1].

Polytrauma is a complex medical condition that is now defined in accordance with the “Berlin definition” as patients who exhibit at least two distinct body regions with an Abbreviated Injury Scale (AIS) ≥ 3, in addition to one or more features selected from the following five physiological parameters: age, consciousness, hypotension, coagulopathy, and acidosis [2]. Prompt interdisciplinary intervention is required for the management of polytrauma, which is distinguished by intricate injuries including fractures, internal hemorrhaging, and head and thoracic trauma [3,4]. This strategy is emphasized through the use of the term ‘golden hour,’ which represents the critical need for early intervention, with a focus on swift stabilization and assessment, to ensure patient survival and recovery [5]. The focus is on rapid assessment of breathing, control of bleeding, and maintenance of cardiovascular stability [6,7].

Approximately 48,000 polytrauma patients are treated in Croatia each year, with 3000 fatalities. 67% of polytrauma cases are caused by road traffic accidents, while falls account for 31%. The average age of polytrauma patients is 40 years. The numbers underscore the persistent difficulty that polytrauma poses to healthcare systems and the crucial requirement for efficient treatment protocols and prevention measures [8].

However, there is no detailed analysis of polytrauma cases treated in Čakovec County Hospital in Croatia. Therefore, the purpose of this study was to determine the number of polytrauma cases at this single-center County Hospital in Croatia, analyze the results, and evaluate the provided therapeutic approaches.

## 2. Materials and Methods

This retrospective cohort study analyzed data of polytrauma patients treated in the emergency department of Čakovec County Hospital, Čakovec, Croatia between 1 January 2019, and 31 December 2022.

### 2.1. Patients

Čakovec County Hospital offers secondary hospital services to the local community and represents a crucial secondary care provider for almost 105,000 residents. Of 104,992 patients treated at the Emergency Department of the Čakovec County Hospital over this four-year period 54 patients met the criteria for polytrauma (as per aforementioned Berlin Definition) [2], and were included in our study. The study was approved by The Ethics committee of the Čakovec County Hospital (approval nr. 01-671/1/2023, 21 February 2023.).

### 2.2. Study Design

To analyze etiology of polytrauma and possible risk factors that led to it, as well as demographic and clinical features of patients, we analyzed electronic and paper medical records of 54 polytrauma patients included in this study. We collected general patient information such as gender, age, as well as ten trauma-related factors including mechanism of injury, blood alcohol level at the time of presentation in ED (tested in certified laboratory using spectrophotometric enzymatic method based on the measurement of NADH produced after addition of alcohol dehydrogenase; blood alcohol levels legally allowed in Croatia ≤ 0.05‰), treatment methods, calendar quarter of injury occurrence, length of hospital stay, and discharge status. Additionally, we recorded triage category at the admission in the ED, and the consciousness level. The triage categories were as follows: category 1—immediate threat to life, category 2—high, but not immediate threat to life, and category 3—non-urgent situations [9]. The consciousness level was assessed using Glasgow Coma Scale score, and we categorized our patients in three groups: GCS 3–8—severe impairment, 9–13—moderate impairment, and 14–15—mild or no impairment [10].

### 2.3. Statistical Analyses

All data are presented as number of patients in a specific category. The relationship between categories (e.g., mechanism of injury and clinical feature) were explored by comparing frequences is given categories using the χ^2^-test. All statistical analyses were done using IBM SPSS Statistics 25. *p* values < 0.05 were considered statistically significant.

## 3. Results

A retrospective analysis was conducted on 54 polytrauma patients who were treated at Čakovec County Hospital from 2019 to 2022 year.

### 3.1. Baseline Characteristics and Seasonal Distribution of Polytrauma Patients

The findings revealed that polytrauma occurrences has seasonal trend: almost two third of polytrauma cases occurred in spring and summer (April–September), while less cases occurred in winter months (October–March), reaching a peak in the third quarter (48%, Table 1). Moreover, there was a clear gender distribution: male patients had a higher prevalence of polytrauma (76%), while female patients accounted for 24% of the cases (Table 1). In addition, the polytrauma was the most frequent in individuals aged 60 years and older (37%, Table 1).

Upon admission to the Emergency Department, all patients underwent assessment of their level of consciousness and were assigned a triage category. The consciousness assessment revealed that 67% of patients had minor or no impairment of consciousness (GCS 14–15), 13% had moderate impairment (GCS 9–13), while 20 had sever impairment of consciousness (GCS 3–8) (Table 1). On the other hand, the triage assessment categorized most patients into category 2 (54%), while smaller portions of patients were classified into category 1 (20%), and category 3 (13%) (Table 1). Surgical intervention was required in 52% of cases, while other were treated conservatively (Table 1).

Laboratory assessment of blood alcohol levels was done in 78% of our polytrauma patients, and only these patients were included further analyses regarding alcohol intoxication.

### 3.2. Mechanism of Injury and Outcome of Polytrauma Patients

Among all polytrauma cases, road traffic accidents were identified as the predominant etiological factor, accounting for 50% of the incidents, as was shown in Table 1. Road traffic accidents and falls, especially from heights on building sites, were identified as the primary causes of injuries, making up over 80% of the cases (Table 1). In 78% of patients for blood alcohol level (BAL) was determined in the laboratory, and half of them had BAL more than 0.05 ‰ showing substantial alcohol consumption, with half of them having an average BAL of around 1.6 ‰ (Table 1).

Following treatment, 70% of patients were discharged directly to their homes, whereas 15% required transfer to tertiary hospitals for further management. Three patients (6%) died in the Emergency department, while five patients succumbed during the subsequent course of treatment in the hospital ward. (Table 1). Of 38 patients discharged home alive from our hospital, 55% required an extended hospital stay of 14 days or more, 26% were treated for two weeks, while 19% were discharged within a week after the admission (Table 1). None of the analyzed demographic or clinical characteristics of the patients demonstrated significant variation across the study years. (Table 1).

### 3.3. The Association of Patients’ Gender and Clinical Characteristics of Polytrauma Patients

The age of our patients was not associated with GCS or triage category. Also, their age did not influence treatment method, discharge status, or the length of hospital stay (Table 2).

Conversely, as demonstrated in Table 2, Glasgow Coma Scale (GCS) scores, triage category, and discharge status were found to be significantly associated with patient gender. While almost three quarters of male patients had GCS 14–15, and only 12% had GCS 3–8, 46% of females had GCS 14–15, but the other 46% had GCS 3–8 (*p* = 0.030).

Consistent with GCS, there is a difference in triage category between genders (*p* = 0.039). More than half of women with polytrauma had immediate threat to life (triage category 1), compared to only 27% of men. Moreover, almost two thirds of men (63%) were in triage category 2 compared to 23% of women (Table 3). In concordance with the differences in GCS and triage category between genders, we recorded the gender-based differences in the outcome (*p* = 0.007). Despite the similar proportion of men and women were discharged home (71% vs. 70%, respectively), more women died in Emergency department (3 compared to none of the men) (Table 3).

### 3.4. The Association of Patients’ Blood Alcohol Level and Other Characteristics of Polytrauma Patients

Although blood alcohol levels were not associated with the age of polytrauma patients (*p* = 0.117), there was the association of blood alcohol levels and gender: while more than a half of men (57%) had blood alcohol levels more than 0.05‰, only 14% had alcohol higher alcohol level that allowed in Croatia (*p* = 0.041, Table 3). However, there was no association of blood alcohol levels and GCS (0.712), triage category (*p* = 0.612), treatment method (*p* = 0.641), discharge status (0.494), or length of hospital stay (*p* = 0.765) (Table 2 and Table 3).

### 3.5. The Association of Mechanism of Injury and Other Characteristics of Polytrauma Patients

Mechanism of injury was not associated with patients’ age (*p* = 0.759), gender (*p* = 0.560) or blood alcohol level (*p* = 0.659). Also, it was not associated with level of consciousness (*p* = 0.472) or triage category (*p* = 0.425) (Table 2).

However, in addition to previously mentioned association of triage category with patients’ gender, the triage category was also associated with patients’ level of consciousness (*p* < 0.001, Table 3): However, the triage category was not associated with age (*p* = 0.245, Table 2). Although the treatment method was not associated with patients’ age (*p* = 0.310), gender (*p* = 0.078), mechanism of injury (*p* = 0.151), or triage category (*p* = 0.0746) (Table 2 and Table 3), it was associated with the length of hospital stay (*p* = 0.029). Therefore, 68% of patients who had surgery stayed in hospital for two weeks or more, compared to only 38% of those who did not undergo a surgery (Table 3).

### 3.6. The Association of GCS and Discharge Status of Polytrauma Patients

In addition to the observed association between discharge status and gender, a significant association was also identified between discharge status and the level of consciousness. (*p* = 0.001). Among patients with a GCS score of 14–15, 28 were discharged home, 6 were transferred to tertiary care facilities, and 2 died during hospitalization. In contrast, all 7 patients with a GCS score of 9–13 were discharged home, with no cases of transfer or in-hospital mortality observed in this group. Conversely, among the 11 patients with a GCS score of 3–8, three were discharged home, two were transferred to tertiary care facilities, and six died—three in the Emergency Department and three during hospitalization in the ward. However, no statistically significant association was observed between discharge status and triage category (*p* = 0.131, Table 3). While the length of hospital stay was associated with the treatment method, as was mentioned above, it was not associated with gender (*p* = 0.920), mechanism of injury (*p* = 0.257), GCS (*p* = 0.392), triage category (*p* = 0.081) (Table 2 and Table 3).

## 4. Discussion

This study analyses data from the emergency department of the Čakovec County Hospital in Croatia over a period of four years. A total of 104,992 patients were treated at the emergency department, of which 54 were identified as polytrauma cases. Although there was no statistically significant difference in the number of cases per year, the annual trends showed that the number of polytrauma cases was highest in 2021 and lowest in 2020, a year greatly affected by the COVID-19 pandemic. The annual fluctuations in polytrauma cases may be associated with external factors like road traffic accidents, severe weather conditions, and the pandemic’s limitations on mobility.

The notable disparity between the proportion of male and female patients treated may be attributed to the varying degrees of injury risk associated with male-dominated occupations and activities. Men are frequently overrepresented in areas like construction, manufacturing, and transportation, which inherently have higher risks of accidents and injuries. The construction sector, which is characterized by a high frequency of falls from heights, accidents involving heavy machinery, and on-site car incidents, mostly hires males. Workplace injuries in certain industries are influenced by gender, with the increased risk being linked to the physical requirements of the professions and societal expectations of masculinity that may deter safety measures [11]. These findings reinforce the need for more individualized preventive approaches, where occupational and lifestyle factors are systematically considered to identify high-risk groups and guide personalized interventions.

Road traffic accidents were found to be the main cause of polytrauma, which is in line with general medical trends [12]. This finding emphasizes the need for comprehensive prevention strategies, such as structural improvements to road infrastructure, more stringent traffic regulations, enhanced driver education programs and widespread public awareness campaigns. Various research has shown that public health mass media campaigns can effectively reduce the frequency of road traffic accidents [13,14,15]. However, beyond population-level approaches, our results highlight opportunities for personalized medicine in prevention. Individual risk profiling, which will incorporate age, sex, occupation, behavioral patterns such as alcohol use, and even genetic susceptibility to injury or slower recover, may allow healthcare systems to design interventions that are both more precise and more effective. For example, digital health tools could be leveraged to provide targeted reminders, driving behavior feedback, or tailored rehabilitation plans based on personal risk factors.

Most polytrauma cases were treated in the summer months, which is probably related to increased outdoor activities, tourism, and driving. A significant percentage of patients were intoxicated, indicating the need for better prevention and education about the risks associated with alcohol consumption and participation in risky activities. The analysis showed a higher incidence of alcohol-related polytrauma in men compared to women, indicating a possible link between male alcohol consumption and an increased risk of polytrauma. As only one female patient was found to have consumed alcohol, this suggests that women may be less likely to consume alcohol in scenarios leading to polytrauma. This is an example in which personalized prevention programs could address behavioral differences across genders.

Despite advances in diagnosis and treatment, trauma remains one of the leading causes of disability and death worldwide. Road accidents are the primary contributor to DALYs among people aged 10 to 49 on a global scale, thereby emphasizing a substantial public health concern. This demographic, consisting of individuals in their youth to middle age, is disproportionately impacted by road traffic conditions due to their considerable mobility and participation in it, whether as pedestrians, passengers, or vehicles [4]. It is important to emphasize that polytrauma can affect people regardless of their age or gender [16]. Changes in the population demographics towards an older age group have resulted in a rise in polytrauma cases among older adults, broadening the range of affected age groups [17]. Despite that, the patients’ age was not associated with any of patient characteristic or their clinical feature in our study. In addition to road traffic accidents, falls from height, sports injuries, acts of violence, and accidents at work are also common causes [18]. However, in our study, the mechanism of injury was not associated with any of patient characteristic or their clinical feature. Our findings indicate that female polytrauma patients were in a more critical condition upon hospital admission, as reflected by significantly lower GCS scores and higher triage urgency compared to male patients. This initial severity was further associated with worse outcomes, including a higher mortality rate among women in the Emergency Department, despite similar overall discharge rates between genders. These results illustrate the value of personalized trauma care. Recognizing that female patients may present with greater initial severity could help creating sex-specific triage algorithms, tailored monitoring protocols, or adjusted thresholds for early interventions. Personalized medicine approaches, such as integrating sex-specific physiological responses into trauma scoring systems, could reduce disparities in outcomes.

The results presented in this study were compared to those from other countries. Numerous studies on polytrauma treatment in outpatient and inpatient facilities in various countries, including Germany, the Netherlands, Brazil, and India [3,19,20], indicate a significantly higher percentage of treated polytrauma cases than in the emergency department of the Čakovec County Hospital. The gender distribution of polytrauma patients was consistent with the results from these countries. Traffic accidents, especially motorbike accidents and falls, proved to be the main causes of polytrauma, with speeding and loss of control of the vehicle contributing particularly to fatal motorbike injuries [5].

The average mortality rate for polytrauma patients in these countries is around 25%. However, it should be noted that the quality and organization of healthcare play a central role in treatment outcomes, especially in polytrauma cases. Differences in healthcare systems and quality of care can significantly affect the prognosis for severely injured patients, including those with severe brain injuries. Differences in public health regulations, emergency response systems, and healthcare access could potentially account for the discrepancy in polytrauma incidence between this study and data from other countries. Regional variations in the incidence of polytrauma cases requiring hospital care could be attributed, for example, to faster emergency response times and more efficient trauma care networks producing greater survival rates. Stricter traffic laws and workplace safety standards, for instance, are examples of strict public safety measures that may have contributed to the lower prevalence of polytrauma cases observed in this study. These elements highlight the significance of a comprehensive strategy that incorporates community education, healthcare infrastructure, and public health policy in the prevention and treatment of polytrauma.

In addition, the results of the study regarding the trends associated with alcohol and seasonality in polytrauma cases offer important information for focused interventions. Given the higher frequency of polytrauma in the summer and the important role that alcohol intoxication plays, it would be possible to more effectively plan and customize public health programs to address these risk factors. For instance, raising public awareness of the risks associated with drunk driving and advocating for safety precautions during seasons of high outdoor activity may help lower the incidence of polytrauma occurrences. Together with further advancements in trauma and emergency treatment, this strategic approach to prevention may improve patient outcomes and lessen the financial strain that polytrauma imposes on the healthcare system.

To gain a deeper understanding of the overall impact of polytrauma injuries on individual quality of life, future research efforts need to investigate the long-term outcomes of these patients, including the success rates of both physical and psychological rehabilitation. Furthermore, studies might investigate how well public health campaigns and certain preventative measures perform to lower the prevalence of polytrauma, especially those that are connected to alcohol use and motor vehicle accidents. Comparative studies carried out across various healthcare systems and geographical areas may serve as a guide for global advances in trauma care techniques. This research could offer deeper insights into the variables contributing to variances in polytrauma incidence and outcomes. To assess their potential for improving the early diagnosis and treatment of polytrauma cases, research on the integration of technological breakthroughs in emergency care, such as telemedicine and AI-driven diagnostic tools, is also required.

This work has its limitations, primarily due to small cohort and single recruitment center. Firstly, when analyzing outcomes of polytraumatized patients, we did not take into account pre-hospital part of polytrauma management, but only patients’ characteristics, mechanism of injury, and in-hospital treatment. However, since the main purpose of this study was not to establish the patients’ outcomes in relation to treatment, we reckon that not taking into account pre-hospital part of polytrauma management do not diminish the findings of this study.

Secondly, despite our results showing that female polytrauma patients had significantly lower GCS scores and higher triage urgency, with a higher mortality rate in ED compared to male patients, we did not investigate the reasons for these findings, since it was out of the scope of this study.

The average mortality rate in our study (15%) was lower than in other studies. Although we stipulate that this difference is caused by the differences in the organization of public healthcare system, emergency response systems, health regulations, and healthcare access between Croatia and systems of other countries presented in those studies [3,19,20], we did not investigate the possible causes for this discrepancy, due to the design of this study.

The findings of this study highlight the necessity for multifaceted preventive interventions, including targeted road safety enforcement, alcohol misuse reduction strategies, and heightened public awareness during high-incidence seasons. Furthermore, optimization of trauma management protocols, integration of advanced diagnostic technologies, and intersectoral collaboration in public health policy are imperative to reduce incidence, improve survival, and minimize the long-term functional and socioeconomic burden of polytrauma. Future multicenter research should explore long-term functional recovery and address gender-specific risk profiles to optimize polytrauma prevention and management in Croatia.

## 5. Conclusions

**Figure 1:**
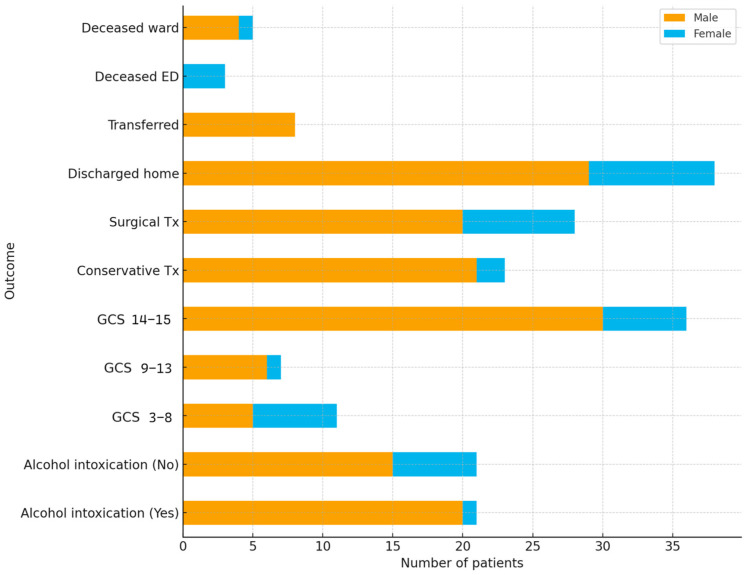
Gender distribution across clinical outcomes.

This four-year retrospective analysis of polytrauma cases at Čakovec County Hospital reveals key epidemiological and clinical patterns relevant to regional trauma care (Figure 1). Most cases involved men, with road traffic accidents as the leading cause, followed by falls, reflecting occupational and activity-related risk profiles, and a seasonal peak in warmer months. Alcohol intoxication was frequent, especially among male patients, highlighting a major modifiable risk factor. Female patients presented with significantly lower Glasgow Coma Scale scores, higher triage urgency, and greater mortality, suggesting sex-based differences in injury presentation. These results indicate potential sex-based differences in injury mechanisms or pre-hospital factors influencing presentation severity (Figure 1).

## Figures and Tables

**Table 1:** The characteristics and clinical features of 54 polytrauma patients in Čakovec County Hospital in Croatia, during four-year period (2019–2022); * χ^2^-test; ^†^ Patients not tested: No. = 12.

Patients’ Characteristics and Clinical Features	No. of Patients				*p* *
	Year				
	2019	2020	2021	2022	
Year period					
January–March	1	4	0	1	0.149
April–June	2	2	4	1	
July–September	7	2	8	9	
October–December	4	3	3	3	
Gender					
Male	9	9	13	10	0.503
Female	5	2	2	4	
Age (years)					
<18	1	0	1	0	0.558
19–29	3	4	2	3	
30–39	0	1	2	1	
40–49	1	0	2	1	
50–59	5	4	2	1	
≥60	4	2	6	8	
Mechanism of injury:					
Fall from height in construction	2	0	2	1	0.109
Other injuries in construction—blunt force trauma	1	1	1	1	
Injuries in road traffic	2	4	13	8	
Fall from height	1	3	4	3	
Injuries caused by firearms	0	1	0	0	
Injuries caused by machinery	0	0	2	0	
Injury inflicted by another person	0	0	3	0	
Injury inflicted by an animal	0	0	1	0	
Alcohol intoxication: ^†^					
No	7	3	5	6	0.104
Yes	5	4	7	5	
Blood alcohol level: ^†^					
0.1–0.5	1	1	1	1	0.754
0.6–1.0	1	0	0	1	
1.1–1.5	1	1	1	2	
1.6–2.0	1	0	0	1	
2.1–2.5	0	2	3	0	
2.6–3.0	1	0	1	0	
3.6–4.0	0	0	1	0	
Glasgow Coma Scale (GCS) score					
3–8	4	2	2	3	0.900
9–13	2	2	1	2	
14–15	8	7	12	9	
Triage category					
1	7	4	2	5	0.093
2	4	5	13	7	
3	3	2	0	2	
Treatment method					
Conservative	4	4	7	8	0.657
Surgery	8	6	8	6	
Discharge status					
Discharged home	8	7	11	12	0.724
Transferred	2	2	3	1	
Deceased at the Emergency Department	2	1	0	0	
Deceased at the hospital ward	2	1	1	1	
Hospital stay (days):					
1–7	0	2	3	2	0.141
8–14	1	2	1	6	
≥15	7	3	7	4	

**Table 2:** The association of some characteristics and clinical features of 54 polytrauma patients in Čakovec County Hospital in Croatia, during four-year period (2019–2022), presented as *p* values (χ^2^-test); * Statistically significant ^†^ Patients not tested: No. = 12.

Patients’ Characteristics	Clinical Features				
	GCS	Triage Category	Treatment Method	Discharge Status	Hospital Stay (Days)
Age	0.098	0.245	0.310	0.702	0.756
Gender	0.030 *	0.039 *	0.078	0.007 *	0.920
Alcohol intoxication ^†^	0.712	0.612	0.641	0.494	0.765
Mechanism of injury	0.472	0.425	0.151	0.151	0.257

OH vs. age: 0.117. Mechanism of injury vs. age: 0.759. Mechanism of injury vs. gender: 0.560. Mechanism of injury vs. OH: 0.659.

**Table 3:** The association of some characteristics and clinical features of 54 polytrauma patients in Čakovec County Hospital in Croatia, during four-year period (2019–2022), presented as *p* values (χ^2^-test); * χ^2^-test; ^†^ Patients not tested: No. = 12.

Patients’ Characteristics	Clinical Features										
	Gender		*p* *	Triage Category			*p* *	Hospital Stay (Days)			*p* *
	Male	Female		1	2	3		1–7	8–14	≥15	
Alcohol intoxication ^†^											
Yes	20	1	0.041	6	12	3	0.612	2	6	9	0.765
No	15	6		9	10	2		2	3	8	
GCS											
3–8	5	6	0.030	11	0	0	<0.001	0	0	3	0.392
9–13	6	1		6	1	0		1	1	5	
14–15	30	6		1	28	7		6	9	13	
Triage category											
1	11	7	0.039	-	-	-		0	1	9	0.081
2	26	3		-	-	-		5	7	11	
3	4	3		-	-	-		2	2	1	
Treatment method:											
Conservative	21	2	0.078	7	12	4	0.746	6	4	6	0.029
Surgery	20	8		8	17	3		1	6	15	
Discharge status:											
Discharged home	29	9	0.007	10	23	5	0.131	-	-	-	
Transferred to hospital ward	8	0		2	4	2		-	-	-	
Deceased at the Emergency Department	0	3		3	0	0		-	-	-	
Deceased at the hospital ward	4	1		3	2	0		-	-	-	

## Data Availability

The original contributions presented in this study are included in the article. Further inquiries can be directed to the corresponding author.
